# Infected Areas around the Ends of Roots of Teeth*Read in the Section on Stomatology of the American Medical Association, at the Sixty-Third Annual Session, held at Atlantic City, June, 1912.

**Published:** 1912-09-15

**Authors:** M. L. Rhein

**Affiliations:** Lecturer on Dental Pathology, Department of Dentistry, University of Pennsylvania, New York


### INFECTED AREAS AROUND THE ENDS OF ROOTS OF TEETH.*

M. L. RHEIN, M.D., D.D.S.

Lecturer on Dental Pathology, Department of Dentistry, University of Pennsylvania, New York.

The spongy character of the alveolar process in which the ends of the roots of the teeth are embedded leaves it especially vulnerable to purulent invasion. This portion of the alveolar process is frequently referred to as the apical space, because so often the osseous structure around the apices of the roots is lost to a greater or less extent on account of an abscess forming in this region. The majority of these infections are alveolar abscesses arising from the death of the pulps of the teeth. There are also pericemental abscesses, existing coincident with living pulps which have not been infected. This latter class may be variously subdivided.

The course of the ordinary alveolar abscess is generally marked by a crisis, at which time the pus escapes into the mouth, either through the plate of the alveolus, or between the periosteum and the root at the gingival border. Unless a radical cure of the abscess is effected a permanent fistulous opening remains, through which afterward a more or less constant flow of pus is discharged into the oral cavity, mixed with the normal fluid and swallowed. This form of abscess is readily diagnosed by reason of the apparent clinical factors, and whatever consequent pathologic lesions result are due to neglect in permitting such an infection to remain *in situ*.

There is, however, another form known as a blind abscess, in which a granulomatous defense seems to aiise, causing a fibrous encystment of the abscessed area. In

*Read in the Section on Stomatology of the American Medical Association, at the Sixty-Third Annual Session, held at Atlantic City, June, 1912.

this case there is no fistula affording an outlet into the mouth. The only symptom is an occasional tenderness over the region of the diseased area, and only too often even this symptom is lacking in bringing attention to the point of infection. Generally this area increases in size and often causes discomfort for the first time after five or ten years of steady encroachment on the contiguous surfaces. This form of abscess is much more dangerous to the individual, because its presence is not suspected although pathogenic conditions may be taking place in various parts of the body as a result of the absorption of these toxins. Although there remains a great amount of work for the bacteriologist in this disease, it is evident that the various forms of streptococci play the predominating role in the same manner that they do in cryptic infections of the tonsils.

The toxemia resulting from these blind abscesses is of such a slow and insidious nature that generally great harm has been done before their presence is suspected. They are a result of a traumatism, some disease of the pulp, or imperfect pulp removal by a dentist. In the last few years the radiograph has demonstrated how few mouths are free from blind abscesses. Gilmer, of Chicago, estimates that 25 per cent. of the people have infected areas around the ends of the roots of their teeth.

The absorption of pus in this manner produces the same results that pus absorption can produce in any other part of the body. Our literature teems with clinical notes of these cases. I could add more data dealing with such conditions, but it is not intended in this presentation to do more than give a synopsis of the factors involved.

In dermatology, the various acnes, eczemas, herpes, erythemas, urticaria, edema, alopecia, seborrhea, psoriasis, erysipelas, etc., can have such an etiologic factor, or their conditions can be complicated by the presence of such septic foci; affections of the upper respiratory tracts, the eye and ear have all been traced to the same source.

Endocarditis and all the allied joint affections frequently owe their inception to a blind abscess. Pernicious anemia has been traced to this source by many authorities. Diseases of the nervous system, even to the production of insanity, have too frequently been cured by the removal of these septic foci to leave any doubt as to the possibilty of their having such dental origin. In like manner clinical data as to nephritis, diabetes, cirrhosis of the liver, and many other diseases caused by infections could be cited to demonstrate the fact that in a correct diagnosis of such contions the possibility of any dental infections should always be considered.

At the present day the radiograph gives us a clear picture of this field and makes a diagnosis comparatively easy, whereas formerly it was not only questionable, but also attended by innumerable obstacles. Superficial mouth examinations by physicians or by incompetent dentists have for many years been the main reason why so many etiologic facts of this nature have not been observed. The true physician cannot continue to salve his conscience by the farce of this kind of oral examination.

The failure of the medical curriculum to give proper stomatologic instruction to the student is primarily the reason why so many forms of malnutrition proceed to an incurable stage before they are even diagnosed. Is it not about time for the American Medical Association to use its power in urging the introduction of such a course in the college curriculum? Only after this shall have been accomplished on a broad and intelligent basis, will this barrier to a more correct diagnosis be destroyed.

If it is true that 25 per cent. of the people have such abscessed areas at the ends of the roots of the teeth, the fact certainly deserves some consideration. A careful investigation of this subject will show that this is not caused by negligence on the part of the people in caring for their teeth, but in most cases is directly traceable to imperfect dental work. If the tooth is to be conserved in a healthy state, after disease and death of the dental pulp, every portion of the organic material in the root canals must be removed and these canals sealed with an impervious homogeneous filling. This operation must be conducted with thorough aseptic precautions so that when it is completed all possibility of future infection shall have been dissipated. The irregularity of many roots and the tortuous nature of some canals make this frequently a very difficult operation and in a small percentage of cases an impossibility. In such cases the infected portion of the root must either be removed or the tooth itself extracted. The imperfect education of dentists is the cause of some of these conditions, but not of the greater majority of them.

The proper removal of such pulp material and the subsequent aseptic sealing of the canal generally entails hours of the most painstaking labor. The average dental practitioner finds it impossible to obtain a living fee for the expenditure of the amount of time necessary in a given case. This has resulted in the practice of a hasty and partial removal of the pulp, and dependence on the insertion in the canals of disinfecting agents to guard against future infection. That such medication has but a temporary value is generally understood; but there is no one to criticize such work.

If any other specialist should leave a portion of necrotic tissue in the body, it would at once bring forth the strongest protest from the patient's regular physician. Nevertheless, dentists are daily performing such surgical operations and leaving portions of necrotic tissue buried in the alveoli to become the foci for future infections. The patient's physician, not only interposes no objection, but likewise submits his own mouth to the same unsurgical procedure. This is no new statement of facts, but it seems that simple words are unavailing in arousing the profession to this continued unnecessary sacrifice of human life. Surely by this time, some little impression should have been made on our confreres. The time must be near at hand when the profession will give this the attention it merits.

ABSTRACT OF DISCUSSION.

Dr. M. H. Fletcher, Cincinnati: I have satisfactorily followed for twenty-five years the practice of putting a very small portion of arsenic, say 0.01 of a grain, as near the apex of these inaccessible roots as possible. It is inserted by winding a few shreds of cotton on the end of a broach and dipping it into the arsenious acid, and then putting it in a root filling. Most persons say this plan is dangerous, but they do not fully comprehend the technic. The whole object of filling the root canal is to keep it aseptic, but if it cannot be obliterated it can be kept aseptic for a lifetime with a little arsenic, because arsenic is so slowly soluble. What little arsenic could get through the apex will easily be taken up by the blood-vessels without injury to the tissues in the apical space. If these spaces are sore from infection they will get progressively worse. Should they become sore from the use of arsenic (which they very rarely do) they will get progressively better. Infection continually increases. Arsenic prevents infection and is continually eliminated. Perfect obliteration of the root canal is the ideal way, but my inability to accomplish this in some cases leads me to endeavor to keep these spaces sterile.

Dr. C. J. Grieves, Baltimore: For the past three years in Baltimore I have been associated with Dr. W. S. Baer of Johns Hopkins University in the study of these conditions, and we have accumulated over a hundred odd cases of this type of infection, apical infection, as the primary portal of entry for infectious arthritis, and in a few cases we have been able to isolate absolutely the infecting micro-organisms; that micro-organism being staphylococcus, contrary to Dr. Rhein's statement—I mean in the type of cases in which we had blind apical abscesses. In almost every instance there had been some bad apical dentistry done—some portion of necrotic tissue left or some effort made to fill the root-canal. The dentist had evidently done all that could be done under the circumstances; he was trying to do an impossible thing in the filling of a tortuous canal and to remove all the contents. Nevertheless, the result was a quiet abscess of which the patient took little or no cognizance, but which had run along for years.

Out of the hundred odd cases there were a few that were clearly straight infections from a tooth-root; general infections were the rule. There would be associated crypts in the tonsils, chronic appendicitis, etc., or some other condition that tended to render this condition possible. These cases were of long standing, with abnormal temperature that ran for months. Some simulated tuberculosis, but most of them were arthritics. The method of diagnosis has been almost entirely that of digital pressure high over the alveolar process. After the area was found a series of small radiographs was taken of not more than the roots of three teeth in any one picture. The only thing we could do to relieve the condition in many cases was extraction. In many fistula cases associated with arthritis (not blind abscesses), when the fistula healed, the temperature of the patient would go up, showing pressure absorption from retention of pus, and when the fistula was opened and drained, the temperature would become normal again. In these cases extraction almost invariably did the work when it was a clear case of the teeth as the primary point of infection.

The medical men with whom I have been associated regularly from the clinical observations condemn a tooth that has a crown on it, so throroughly do they associate these pus conditions with the crowned tooth. Of course, we know as dentists that that is an injustice. They have however, seen so many of these cases in which poor canal work has been done and the teeth crowned and which have later resulted in necrotic conditions in these areas that I regard them as justified in asking for radiographs of the tissues round the apical ends of the crowned teeth.

Dr. M. I. Schamberg, New York: While it may be true that Dr. Fletcher and other members of this Section are active in the instruction of students in medical schools, this practice is far from being general. There is no reason why the student should be ignorant of this subject any more than any other branch of the healing art, and, moreover, the men should be compelled to pass their examinations on this subject, just as they would on the eye, the ear, the nose, the throat and other parts of the body. I do not believe that it is necessary for our Section to enter into a matter which can be so readily discussed elsewhere. I believe that this Section should be active in trying to do something rather than trying to solve something.

Dr. Thomas L. Gilmer, Chicago: The importance of good hygiene of the mouth cannot be overestimated. Oral pathology should be better taught in medical schools. In general pathology physicians are usually well informed, but deficient in oral pathology. I think it would be most damaging to let Dr. Fletcher's statement go unchallenged that it is good practice to put arsenic on cotton in the roots of teeth and depend on it as a permanent antiseptic.

Arsenic has no place in the teeth at all. It will not remain at the end of the roots indefinitely, as an antiseptic. If a medicament is soluble it will not permanently remain in the root; if it is insoluble it is not an antiseptic. The idea of utilizing antiseptics as permanent root fillings is impracticable. The apical ends of some pulpless roots become encysted even if they are not well filled, and such roots will do no harm. Radiographs, on the other hand, show that in some instances well-filled roots have blind abscesses at their apices.

I believe that we extract too few teeth; we used to extract too many. We can, however, in some instances cure chronic alveolar abscesses, which are incurable by medication through root canals, by resection of the offending part of the root and curetting the walls of the abscess.

Dr. S. L. McCurdy, Pittsburgh: The word "infection" has been, I think, used very loosely in this connection. A cyst on the end of the root may become an open cyst, may become infected and become an abscess. When we talk about bacteria on the end of the root of the tooth, the question arises, How did the infection get there?

Dr. E. S. Talbot, Chicago: I do not believe that the profession to-day is aware of the number of peridental abscesses that there are in the mouth. These abscesses lie dormant for years. I had a tooth extracted two weeks ago with a blind abscess on it which I believe to have been in my mouth for fifty-two years. When a boy, 10 or 12 years old, I had a tooth-ache, and a country doctor tried to remove that tooth with the old-fashioned turn-key. He failed to remove the tooth, but he stopped the pain, and from that time to this I have never had any pain in that tooth. The tooth was afterward crowned, and it has been of service to me since until I was obliged to have it removed.

I honestly believe that these abscesses are doing a great deal of damage. I believe that arthritis is the result, but at present we have no direct proof. We know that pus is distributed directly into the blood from these abscesses; we know also that pus is swallowed every time we take food into the mouth. Do pus germs pass through the stomach when hydrochloric acid is present? Of course, hydrochloric acid is present only with digestion of foods. It is possible that these germs can pass through when hydrochloric acid is not there. This has not been really demonstrated. No one has found pus germs in the stomach up to the present time. One man has found pus germs in the feces in some ten or twelve examinations.

Dr. M. L. Rhein, New York: I agree with Dr. Gilmer's criticism of Dr. Fletcher's technic in treatment of root canals, the end of which it is impossible to reach. The theory needs to be supplemented by scientific facts; not clinical data, but proofs that infection is impossible. The fact that Dr. Fletcher has had splendid results from sealing an infinitesimal amount of arsenic in the end of the canal is, to my mind, no proof that subsequent infection will not take place. If Dr. Fletcher will have a large number of such teeth on which he operated in years past radiographed it will give us an opportunity to make a reasonable scientific deduction as to the results.

I do not agree with Dr. McCurdy that it is a complicated point as to the source of infection in this area. There are only two methods by which infection of these areas can be obtained: either through the mouth arising from the defects in the technical work of sealing the root canals and absorption of bacteria, or through the circulation at the end of the root. I am convinced that such infections as we have commonly looked on as the worst, in which there was an open fistula from the abscess with the patient swallowing pus in large quantities, was not nearly so detrimental to the patient as the little blind abscess at the end of the root. There is no question but that certain secretions in the intestinal tract destroy a portion of the swallowed pus. A root canal may be imperfectly filled and go for many years without any infection. I question the statement Dr. Talbot made in reference to the tooth in his own mouth, that this blind abscess had been attached to that root for fifty-two years. It may be that this abscess only appeared within the past few years. I have examined root canals that I have filled years before we had the radiograph in which I thought at the time that I had reached the ends of the roots, and have found that the filling did not go to the very end of the canal. The space we speak of as the apical space was, however, in an absolutely physiologic condition. When Dr. Gilmer speaks of improper dental work resulting in the death of many people, he has not exaggerated one iota. If pulp canal work is done it is essential that the aseptic filling material should go to the very end of the canal if we want to have absolute assurance that secondary infection through the circulation cannot take place.—*Journal A. M. A.*, August 3d, 1912.

